# Destination and Specific Impact of Different Bile Acids in the Intestinal Pathogen *Clostridioides difficile*

**DOI:** 10.3389/fmicb.2022.814692

**Published:** 2022-03-24

**Authors:** Nicole G. Metzendorf, Lena Melanie Lange, Nina Lainer, Rabea Schlüter, Silvia Dittmann, Lena-Sophie Paul, Daniel Troitzsch, Susanne Sievers

**Affiliations:** ^1^Department of Pharmacy, Uppsala University, Uppsala, Sweden; ^2^Department of Microbial Physiology and Molecular Biology, Institute of Microbiology, Center for Functional Genomics of Microbes, University of Greifswald, Greifswald, Germany; ^3^Imaging Center of the Department of Biology, University of Greifswald, Greifswald, Germany

**Keywords:** *Clostridioides difficile*, bile acids, toxin A, flagella, adhesion

## Abstract

The anaerobic bacterium *Clostridioides difficile* represents one of the most problematic pathogens, especially in hospitals. Dysbiosis has been proven to largely reduce colonization resistance against this intestinal pathogen. The beneficial effect of the microbiota is closely associated with the metabolic activity of intestinal microbes such as the ability to transform primary bile acids into secondary ones. However, the basis and the molecular action of bile acids (BAs) on the pathogen are not well understood. We stressed the pathogen with the four most abundant human bile acids: cholic acid (CA), chenodeoxycholic acid (CDCA), deoxycholic acid (DCA) and lithocholic acid (LCA). Thin layer chromatography (TLC), confocal laser scanning microscopy (CLSM), and electron microscopy (EM) were employed to track the enrichment and destination of bile acids in the bacterial cell. TLC not only revealed a strong accumulation of LCA in *C. difficile*, but also indicated changes in the composition of membrane lipids in BA-treated cells. Furthermore, morphological changes induced by BAs were determined, most pronounced in the virtually complete loss of flagella in LCA-stressed cells and a flagella reduction after DCA and CDCA challenge. Quantification of both, protein and RNA of the main flagella component FliC proved the decrease in flagella to originate from a change in gene expression on transcriptional level. Notably, the loss of flagella provoked by LCA did not reduce adhesion ability of *C. difficile* to Caco-2 cells. Most remarkably, extracellular toxin A levels in the presence of BAs showed a similar pattern as flagella expression. That is, CA did not affect toxin expression, whereas lower secretion of toxin A was determined in cells stressed with LCA, DCA or CDCA. In summary, the various BAs were shown to differentially modify virulence determinants, such as flagella expression, host cell adhesion and toxin synthesis. Our results indicate differences of BAs in cellular localization and impact on membrane composition, which could be a reason of their diverse effects. This study is a starting point in the elucidation of the molecular mechanisms underlying the differences in BA action, which in turn can be vital regarding the outcome of a *C. difficile* infection.

## Introduction

The intestinal pathogen *Clostridioides difficile* is a strictly anaerobic bacterium and causes one of the most frequent hospital-acquired infections in developed countries called CDI for *Clostridioides difficile* infection. *C. difficile* produces two major toxins (toxins A and B) that provoke diarrhea in the first instance (Thomas et al., 2003), and in more advanced and serious cases pseudomembranous colitis, toxic megacolon and possibly even an intestinal perforation leading to sepsis (Rupnik et al., 2009). The bacterium is capable of forming extremely resistant spores that persist antibiotic treatment resulting in very high relapse rates. It is commonly appreciated that an intact intestinal microbiota can prevent bacterial infections, a phenomenon which was called colonization resistance (Buffie and Pamer, 2013). A disturbed intestinal microbiota (dysbiosis), as it can be found after antibiotic treatment, constitutes a welcome opportunity for bacterial pathogens to proliferate in the intestinal tract. In fact, systemic antibiotic treatment and subsequent dysbiosis represent the major risk factor to develop a CDI (Reeves et al., 2011). However, the mechanisms behind the microbiota-mediated colonization resistance are only poorly understood. It is assumed that the structural rearrangements in the microbiota after antibiotic treatment come along with functional and thus metabolic changes creating a basis for germination, growth and toxin production of *C. difficile* (Theriot and Young, 2014, 2015). In 2014, Theriot et al. (2014) could substantiate such metabolic alterations in mice and showed that antibiotic treatment led to an increase of primary bile acids (PBAs), which are produced by the human liver. Concurrently, a decrease in the levels of secondary bile acids (SBAs), which are formed from PBAs by the microbiota was detected. BAs are also thought to be one of the main reasons for the success of fecal microbiota transplantations (FMTs) applied in very heavy cases of CDI (Cammarota et al., 2014). It could be demonstrated that *via* FMT a dysregulated BA composition of CDI patients could be corrected to a composition that suppresses germination of *C. difficile* spores and inhibits growth of the pathogen (Weingarden et al., 2016).

Bile acids are amphiphilic compounds with a steroid structure produced from cholesterol in the liver and secreted into the intestine. They facilitate the absorption and digestion of dietary lipids, but also represent natural antimicrobials due to their soap-like character (Begley et al., 2005). The PBAs cholic acid (CA) and chenodeoxycholic acid (CDCA) are mostly conjugated to taurine or glycine. After deconjugation by specific species of the microbiota in a first step, they can be further converted to the SBAs deoxycholic acid (DCA) and lithocholic acid (LCA), respectively, by dehydroxylation at C7. Along the intestinal tract, the composition of BAs changes due to the conversion of PBAs to SBAs by species of the microbiota, and the total concentration of BAs constantly decreases with passage through the intestines, since up to 95% of BAs are re-absorbed by intestinal epithelial cells (Hofmann, 2009). Only during the last years the key players in the complex human microbiota, that are capable of modifications of BAs, could be pinpointed (Theriot and Young, 2014). Specifically, the ability of *C. scindens* of a 7-alpha-dehydroxylation on the steroid body to form SBAs enhances resistance to CDI (Buffie et al., 2015). However, SBAs are a double-edged sword, since they do not only protect from CDI but are also associated with some diseases such as gallstone formation and colon cancer (Ridlon et al., 2016). In light of this fact, it is of utmost importance to understand the action of BAs on a molecular level, before they or analogs of them could be considered as drugs for treating and preventing CDI.

Membrane-damaging is the most reported effect of BAs on bacteria which could be attributed to their amphiphilic nature. However, at lower concentrations the effect of BAs can be more subtle as minor changes in the membrane do not result in loss of cell integrity but rather in changes in membrane fluidity, transmembrane fluxes or activity of membrane-bound enzymes (Begley et al., 2005). *Vice versa*, stress-mediated alterations in membrane architecture and composition, e.g., by temperature, significantly influence BA resistance (Fernández Murga et al., 1999). All known sublethal stresses that provide cross-protection to BAs are known to induce membrane changes (Begley et al., 2005). In addition to the membrane-disturbing effect, BAs were also reported to structurally alter other macromolecules as RNA, DNA and proteins (Begley et al., 2005). LCA was even reported to cause single strand breaks in DNA in mouse lymphoblasts (Kulkarni et al., 1980).

Bile acid stress experiments in bacteria were mostly carried out using a complex BA mixture isolated from ox or pig (Begley et al., 2002). Thus, very few specific BAs effects are reported and almost nothing is known on the signal transduction of the BA stimulus, e.g., if it is sensed by a two-component system, by a specific receptor or merely indirectly by disruption of membrane integrity or BA-induced alterations in the structure of macromolecules. However, reports on contrary responses to BAs by different species hint at specific receptors and signal transduction pathways. For instance, BAs have been shown to upregulate the flagellar promoter in *Campylobacter jejuni* (Allen and Griffiths, 2001) and motility in *Vibrio cholerae* (Krukonis and DiRita, 2003), but in *Salmonella*, flagellar synthesis and motility was reduced in the presence of BAs (Prouty et al., 2004).

Regarding the action of BAs on *C. difficile*, some specific effects have been described. Already decades ago, Wilson reported a stimulation of spore germination by specific bile preparations (Wilson, 1983). In 2008 CA was elucidated to be the active bile component that instigates spore germination (Sorg and Sonenshein, 2008) and eventually, the protease CspC on the spore surface was demonstrated to be the receptor (Francis et al., 2013) for taurocholate which initiates germination. CspC is the only direct BA receptor in *C. difficile* so far contrasting the human host, where far-reaching effects of BAs due their specific binding to diverse BA receptors (Fiorucci and Distrutti, 2015) have been recognized. BAs do not only act on *C. difficile* spores. An inhibitory effect of SBAs on growth and virulence of *C. difficile* has been frequently described (Lewis et al., 2016; Winston and Theriot, 2016; Thanissery et al., 2017). Interestingly, also a restraining action of BAs on the toxins has been found pointing at a possible direct interaction of BAs and *C. difficile* toxins (Brandes et al., 2012; Darkoh et al., 2013). Lewis et al. (2016) found that *C. difficile* strains that exhibit a high tolerance to LCA *in vitro* are more virulent in animals and very recently, DCA was confirmed to promote biofilm formation in *C. difficile* (Dubois et al., 2019). Furthermore, Kang et al. (2019) reported an increased inhibitory effect of tryptophan-derived antibiotics on *C. difficile* in the presence of DCA and LCA but not CA. The dissimilar impact of various BAs in *C. difficile* is reflected by the difference of proteomic stress signatures as described recently (Sievers et al., 2019).

In order to understand and to anticipate the susceptibility of patients to develop a CDI as well as to consider BA analogs for treatment or prevention of this infectious disease, more precise knowledge on the targets and mode of action of BAs in *C. difficile* has to be attained. In this study, the effect of four infection-relevant BAs on important virulent traits of *C. difficile* has been characterized. We could show a reduction of flagella in the presence of selected BAs and could ascribe the origin of reduced flagella to transcriptional level. The similar effect could be demonstrated for the abundance of secreted toxin A. However, adhesion to Caco-2 cells did not follow the same pattern and seems not to be exclusively dependent on flagella. Tracking the destination of the four BAs revealed a high accumulation of LCA in *C. difficile* and a distinct distribution of the other BAs in the *C. difficile* cell. In summary, the results do not only disclose specific and infection-relevant novel phenotypes of *C. difficile* in response to various BAs, but also point at the different target location of BAs in the cell to be one of the reasons for the specific phenotypes.

## Materials and Methods

### Strains and Growth Conditions

Strains R20291 and 630 of *C. difficile* obtained from the “German Collection of Microorganisms and Cell Cultures GmbH” (DSM27147 and DSM27543, respectively) were used in this study. For long-term (LT)-stress experiments, *C. difficile* was inoculated to an A_600_ of 0.01 in brain heart infusion medium (BHI, Oxoid Limited, Hampshire, United Kingdom) and grown anaerobically at 37°C (Don Whitley Scientific Limited, Herzlake, Germany) in the presence of growth-inhibiting concentrations of the sodium salts of the four main bile acids (BAs), which are cholic acid (CA) at 15 mM, chenodeoxycholic acid (CDCA) at 0.8 mM, deoxycholic acid (DCA) at 0.8 mM and lithocholic acid (LCA) at 0.08 mM. All BAs were obtained from Sigma Aldrich (Taufkirchen, Germany) except for LCA which was from Steraloids Inc. BA treated cells and untreated cells were harvested anaerobically at exponential growth phase (*A*_600_ = 0.4–0.6) and at stationary phase (*A*_600_ = 1.0–1.2 for stressed cells, >2.0 for untreated cells and cells stressed with LCA).

### RNA Preparation and Northern Blot Analysis

Bacterial suspension was harvested under anaerobic conditions and collected in ice-cold falcon tubes. The samples were quickly cooled down by placing them carefully in liquid nitrogen. Pelleting of cells was done by centrifugation at 10,200 × *g* at 4°C for 3 min. The supernatant was discarded, the pellet dissolved in sterile suspension buffer (3 mM EDTA, 200 mM NaCl, pH 8.0) and after mechanical disruption proceeded with an acid-phenol extraction of RNA (Nicolas et al., 2012). Quality of the RNA was analyzed on a 1.5% agarose gel and concentration was determined with NanoDrop DeNovix DS-11 Spectrophotometer (DeNovix, Wilmington, United States). RNA was extracted in quadruplicates from independent cultures.

Northern blot analysis was carried out as previously described (Troitzsch et al., 2021). The digoxigenin-labeled RNA probes were synthesized by *in vitro* transcription with T7 RNA polymerase using gene-specific primers (fliC-NB forw 5′-ATGAGAGTTAATACAAATGTAAGTGC-3′ and fliC-NB rev 5′-CTAATACGACTCACTATAGGGAGACTATCCTAATAATTGT AAAACTCC-3′) on chromosomal DNA as template. 10 μg of total RNA were separated on a 1.5% agarose gel per sample. Chemiluminescent signals were detected with a Lumi-Imager (Intas Science Imaging Instruments GmbH). Intensity signals of Northern Blot bands were quantified with Fiji (Schindelin et al., 2012).

### Purification of Cell Surface Proteins and Western Blot Analysis

Cell envelope proteins were purified as described earlier by Delmée et al. (1990) and Twine et al. (2009) with a few adaptations (Delmée et al., 1990; Twine et al., 2009). In brief, 100 μL of a *C. difficile* 630 overnight culture were plated on BHI agar plates containing the four BAs at concentrations used for LT-stress and the plates were incubated at anaerobic conditions at 37°C for 48 h. The bacterial lawn was harvested by adding 2 mL water and carefully scraped off with a plastic inoculation loop. The suspensions were intensely shaken on a vortex mixer at maximum power for 3 min followed by centrifugation at 13,500 × *g* at 4°C for 5 min. The supernatants were lyophilized, resuspended in 300 μL water and desalted by using Micron^®^centrifugal filter devices (Amicon) at 14,000 × g at 4°C for 20 min. Protein concentration was determined using Roti^®^Nanoquant (Carl Roth, Karlsruhe, Germany). 10 μg of each protein sample was separated on a 10% SDS-PAGE (Amplichem) at constant voltage of 80 V. Gels were stained with Coomassie Brilliant Blue R (Sigma Aldrich, Taufkirchen, Germany) or for Western Blot analysis blotted onto a PVDF membrane (Merck) for 90 min at 100 V. The membrane was incubated for 1 h in Tris-buffered saline (TBS; 10 mM Tris, 150 mM NaCl, 20 mM NaN_3_; pH 8.0) buffer containing 0.1% (v/v) Tween-20 (Serva, TBST) and 5% (w/v) skimmed milk powder (Carl Roth, Karlsruhe, Germany). After three washing steps in TBST, the first antibody (chicken anti-FliC *C. difficile* IgY kindly provided by Glen Armstrong) 1:20,000 (v/v) and rabbit anti-FliD *C. difficile* (kindly provided by Severine Pechine) 1:1,000 (v/v) was added in TBST with 5% (w/v) skimmed milk powder and incubated overnight at 4°C. The membrane was washed three times in TBST, before incubation with the secondary antibody (anti-chicken alkaline phosphatase (AP)-conjugated (Sigma Aldrich, Taufkirchen, Germany) 1:10,000 (v/v), anti-rabbit AP-conjugated (Sigma Aldrich, Taufkirchen, Germany) 1:10,000 (v/v) diluted in TBST, 5% (w/v) skimmed milk powder for 45 min at room temperature (RT). The membrane was washed 3 times in TBST followed by another wash step with ice-cold alkaline phosphatase AP-buffer (0.1 M Tris–Hcl, 0.1 M NaCl, pH9.5). The signal was detected by adding NBT (5% p-nitroblue tetrazolium chloride in 70% (w/v) dimethylformamide) and BCIP [5% 5-bromo-4-chloro-3-indolyl phosphate in 100% dimethylformamide (w/v)] in a ratio of 2:1 (v/v) in AP-buffer. The reaction was stopped with H_2_O. Intensity of Western Blot signals was quantified using Fiji (Schindelin et al., 2012).

### Eukaryotic Cell Culture

Co-cultivation of *C. difficile* with the human colon carcinoma cell line Caco-2 (ATCC No. HTB-37) was based on a previously described protocol (Natoli et al., 2012). Cells were routinely grown in Dublecco’s Modified Eagle’s Medium (DMEM, Gibco, Thermo Fisher Scientific) supplemented with 10% inactivated fetal calf serum (FCS) (Sigma Aldrich, Taufkirchen, Germany) and 1% MEM non-essential amino acids (Gibco) in a 5% CO_2_ atmosphere at 37°C. Confluency occurred after 5–6 days of incubation. For the adhesion assay, propagated cells were seeded at 5 × 10^4^ per well onto pre-cleaned and poly-L-lysine-coated (PLL; Sigma, 1:10 (v/v) in deionized water) 10 or 18-mm-diameter high precision coverslips (Paul Marienfeld GmbH, Lauda-Königshof, Germany) in a 12-well tissue culture plate (TPP Techno Plastic Products AG, Trasadingen, Switzerland). The culture medium was changed every other day. Cells were used 7 days (almost confluent monolayers) after seeding. The state of polarization and morphological differentiation was monitored by scanning electron microscopy (SEM).

### Adhesion Assay

*C. difficile* adhesion on Caco-2 cells was assayed according to an adapted protocol from Cerquetti et al. (2002). Caco-2 cells were carefully washed with PBS and kept in DMEM without phenol red (Gibco) supplemented with 10% FCS in a 5% CO_2_ atmosphere at 37°C until co-cultivation. Bacterial cells were harvested by centrifugation at 630 x *g* for 10 min at RT in air-tight falcons (TPP Techno Plastic Products AG, Trasadingen, Switzerland), washed once with phosphate buffered saline (PBS; 137 mM NaCl, 2.7 mM KCl, 1.4 mM KH_2_PO_4_, 10 mM Na_2_HPO_4_ × 2 H_2_O; pH 7.4) and diluted 1:10 (v/v; 1 × 10^8^ bacteria mL^–1^) in DMEM without phenol red supplemented with 10% FCS. 1 mL of these bacterial suspensions was added to each tissue-culture plate well and incubated for 2 h anaerobically. The bacterial cell suspension was carefully removed and cells were washed 3 times with sterile anaerobic PBS and finally incubated with 1% (v/v) Triton X-100 (Serva) for 5 min at 37°C within the anaerobic chamber. The suspension was removed and dilutions were plated on pre-equilibrated BHI agar plates (Oxoid Limited, Hampshire, United Kingdom). After 24 h the counts of adherent bacteria released from Caco-2 cells were determined. The results were expressed as number of adherent bacteria per cell. For this purpose, Caco-2 cells from a number of non-infected monolayers were collected by trypsinization and counted in parallel. Each experiment was carried out in technical triplicates of three independent experiments. For scanning electron microscopy (SEM), infected monolayers were grown on 10-mm-diameter high precision coverslips (Paul Marienfeld GmbH, Lauda-Königshof, Germany), which were fixed in the medium with 2% (v/v) glutaraldehyde (GA, Sigma Aldrich, Taufkirchen, Germany) and 5% (v/v) paraformaldehyde (PFA, Science Services GmbH, Munich, Germany) for 30 min at RT followed by an incubation at 4°C overnight. The coverslips were then transferred in a 12-well dish containing fixative No. 2 (100 mM HEPES, 50 mM NaN_3_, 2% (v/v) GA, 5% (v/v) PFA) and stored at 4°C until further processing.

### Confocal Laser Scanning Microscopy

Infected monolayers of Caco-2 cells were fixed with 4% (v/v) PFA in PBS for 12 min in the anaerobic chamber. *C. difficile* in suspension were either pelleted at 160 × g for 5 min and 4% (v/v) PFA was added to the pellet for 12 min at RT or added to PLL-treated 18-mm-diameter coverslips (Paul Marienfeld GmbH, Lauda-Königshof, Germany) and incubated to attach for approx. 30 min proceeded by fixation with 4% (v/v) PFA under anaerobic conditions. All cells were washed for 5 min with PBS thrice and optionally permeabilized with 0.25% (v/v) Triton-X-100 (Serva) for 5 min at RT and carefully rinsed with PBS. Blocking was done with 0.5% (w/v) BSA (Sigma Aldrich, Taufkirchen, Germany) for 1 h at RT. *C. difficile* were labeled using rabbit anti-*C. difficile* No. 501 diluted 1:1,000 (v/v), (provided by Rob Fagan), followed by incubation with the secondary goat anti-rabbit IgG (H + L) Alexa Fluor 488 antibody [1:1,000 (v/v), Thermo Fisher Scientific].

For preparation of sections, cells were fixed as described for transmission electron microscopy (see below). The following day, samples were centrifuged at 1,500 × *g* for 5 min, the pellet was washed with washing buffer (100 mM cacodylate buffer; pH 7.4) thrice for 5 min each and after a final centrifugation step at 9,600 × *g*, the pellet was embedded in low gelling agarose. The material was washed with washing buffer three times for 10 min at RT, dehydrated in graded series of ethanol (30, 50, 70, 90, and 100% for 30 min each step on ice) and subsequently infused with the acryl resin LR White (Plano GmbH, Wetzlar, Germany). Embedding and the following steps were carried as described by Petersen et al. (2020) for sample preparation for serial sectioning and immunofluorescence labeling.

Bile acids were each detected with a 1:100 (v/v) dilution of mouse anti-CA (Dianova), mouse anti-CDCA (Dianova), rabbit anti-DCA (Cloudclone Corp., Hölzel Diagnostika, Köln, Germany) and rabbit anti-LCA (Hölzel Diagnostika, Köln, Germany) followed by incubation with a goat anti-mouse IgG (H + L) Alexa Fluor 546 (Invitrogen) or goat anti-rabbit IgG (H + L) Alexa Fluor 488 (Thermo Fisher Scientific) secondary antibody, respectively, both diluted 1:1,000 (v/v). Caco-2 cells were visualized with 1:1,000 (v/v) Rhodamine-phalloidine stain Alexa Fluor 568 (Thermo Fisher Scientific). DNA was labeled with 1:1,000 (v/v) of 4′,6-diamidino-2-phenylindole (DAPI, Thermo Fisher Scientific) and the membrane visualized with a dilution of 1:1,000 (v/v) Nile red (Thermo Fisher Scientific). All antibody dilutions were prepared in 0.5% (w/v) BSA in PBS and cells were incubated for 2 h with the first antibody and 45 min with the secondary. After antibody incubation, cells were always washed 3 times for 5 min with PBS and once with water before mounting in Mowiol (Carl Roth, Karlsruhe, Germany). Fluorescence images were acquired using an inverted laser scanning confocal microscope (Zeiss LSM 510 Meta, Carl Zeiss Microscopy GmbH) equipped with a 100 × oil objective (NA 1.4) and the ZEN 2009 software (Carl Zeiss Microscopy GmbH). An argon/krypton laser (488/568 nm) as well as a HeNe-laser (633 nm) were used at 25% intensity. A diode laser (405 nm) was used at 15% intensity. The excitation and emission wavelengths employed were 488 and 510 nm for Alexa Fluor 488, 532, and 570 nm for Alexa Fluor 546 and 568 and 590 nm for Alexa Fluor 568, respectively. Images were captured using a 512 × 512-pixel frame. Gain settings were between 600 and 800. Scan speed was set to 7 and the mean of 4 lines was used. The zoom function and airy unit was set to one. Z-series were used to capture out of focus filaments.

### Scanning Electron Microscopy

*C. difficile* bacterial suspensions were fixed in the medium with 2% (v/v) GA (Sigma Aldrich, Taufkirchen, Germany) and 5% (v/v) PFA (Science Services GmbH, Munich, Germany) for 30 min at RT followed by an incubation at 4°C overnight. Cells were subsequently added on PLL-treated coverslips and left to attach in a humid chamber for approx. 60 min at RT. The remaining liquid was carefully removed, the coverslips were transferred into a 12-well dish containing fixative No. 2 (see above) and stored at 4°C. All coverslips (with single cells or with infected monolayers) were washed with washing buffer (100 mM cacodylate buffer, 1 mM CaCl_2_; pH 7) three times for 5 min each. Then, the samples were dehydrated in a graded series of aqueous ethanol solutions (10, 30, 50, 70, 90, and 100%) on ice for 15 min each step. Before the final change of 100% ethanol, the samples were allowed to reach room temperature and then critical point-dried with liquid CO_2_. Finally, samples were mounted on aluminum stubs, sputtered with gold/palladium and examined with a scanning electron microscope EVO LS10 (Carl Zeiss Microscopy Deutschland GmbH, Oberkochen, Germany). All micrographs were edited by using Adobe Photoshop CS6. Scanning electron micrographs were analyzed with Fiji (Schindelin et al., 2012) to determine the length of the cells (stationary phase: CA *n* = 37, DCA *n* = 61, CDCA *n* = 58, LCA *n* = 55, untreated *n* = 65). All data were further analyzed using Prism (version 9.2.0). The average was calculated and the standard deviation is indicated. Statistical significance was assessed by an unpaired Student’s *t*-Test (***p < 0.001).

### Transmission Electron Microscopy

The used Transmission Electron Microscopy (TEM) procedure is adapted to a protocol kindly provided by Aimee Shen.

*C. difficile* cells were pelleted in air-tight and pre-equilibrated Falcon tubes (TPP Techno Plastic Products AG, Trasadingen, Switzerland) by centrifugation at 4,000 x *g* for 10 min at 4°C. Cells were washed once with sterile-filtered (0.2 μm) PBS and transferred to 1.5 mL SafeSeal Microcentrifuge Tubes (Sorenson™ BioScience Inc., Allentown, PA, United States) to repeat the centrifugation step. The supernatant was discarded and the pellet was resuspended in PBS and fixative (100 mM cacodylate buffer, 2% (v/v) PFA, 2% (v/v) GA, 5 mM CaCl_2_, 10 mM MgCl_2_, 50 mM NaN_3_; pH 7.4) in a ratio of 1:5 (v/v). The samples were incubated for 10 min at 20°C and then shifted to 4°C slightly nutating overnight. The following day, samples were centrifuged at 1,500 × *g* for 5 min and the pellet was washed with washing buffer (100 mM cacodylate buffer; pH 7.4) three times for 5 min each. After a final centrifugation step at 9,600 × *g* the pellet was embedded in low gelling agarose. Cells were postfixed in 1% osmium tetroxide in washing buffer for 1 h at 4°C, and then stained with 0.5% uranyl acetate in 0.9% (w/v) sodium chloride at 4°C overnight with washing steps in between. After dehydration in graded series of ethanol (35 – 100% ethanol), the material was transferred stepwise into propylene oxide and finally embedded in AGAR-LV resin (Plano, Wetzlar, Germany). Sections were cut on an ultramicrotome (Reichert Ultracut, Leica UK Ltd., Wetzlar, Germany), stained with 4% aqueous uranyl acetate for 5 min followed by lead citrate for 1 min and analyzed with a transmission electron microscope LEO 906 (Carl Zeiss Microscopy Deutschland GmbH, Oberkochen, Germany). All micrographs were edited by using Adobe Photoshop CS6.

### Preparation of Extracellular Proteins for Toxin A Western Blot Analysis

*C. difficile* R20291 and 630 were anaerobically cultivated in tryptone medium (3% (w/v) tryptone, 2% (w/v) yeast extract, and 0.12% thioglycolic acid) at 37°C in three biological replicates. Cultures were inoculated to an A_600_ of 0.05, treated with growth-inhibiting concentrations of the sodium salts of CA, CDCA, DCA, and LCA as previously described (Sievers et al., 2019) and cultivated for 72 h. Subsequently, 20 mL of each culture was centrifuged at 10,200 × *g* for 20 min at 4°C. The supernatant was transferred into a new centrifuge tube and precipitated with 10% (v/v) trichloroacetic acid overnight at 4°C. Thereafter, precipitated extracellular proteins were pelleted *via* centrifugation for 1 h at 10,200 × *g* at 4°C. Pellets were washed in 1 mL 70% (v/v) ethanol, transferred to a new reaction tube and centrifuged at 11,000 × *g* for 5 min at 4°C. The washing step was repeated twice with 100% ethanol. Pellets were dried and subsequently dissolved in 100 μL urea buffer (8 M urea, 10 mM EDTA, 200 mM EDTA, 1% (w/v) CHAPS). Protein concentration was determined using Pierce™ BCA Protein Assay Kit (Thermo Fisher Scientific) following the manual for the “enhanced protocol.”

In total, 150 μg of each protein sample was separated *via* SDS-PAGE. The gel was run at 70 V until samples migrated into the 8% separating gel und was then run at 100 V until samples passed the complete gel. Subsequently, proteins were blotted onto polyvinylidene fluoride (PVDF) membranes (Merck Millipore, Darmstadt, Germany) for 2 h at 100 V. Blotted membranes were treated and detected as described above, but using a primary antibody against TcdA (1:10,000 (v/v), tgcBiomics, Bingen, Germany) and an anti-mouse antibody as secondary antibody (1:25,000 (v/v), Sigma Aldrich, Taufkirchen, Germany) Blots were scanned and signals were quantified using ImageJ (Schneider et al., 2012).

### Thin Layer Chromatography

Lipids were extracted according to an adapted protocol by Nakayasu et al. (2016). Briefly, bacterial cells were pelleted by centrifugation at 10,200 x *g* for 10 min at 4°C. The supernatant was discarded and the pellets were resuspended in freshly prepared 150 mM ammonium bicarbonate and subjected to vortex mixing for 30 sec. A volume of four-fold excess of pre-warmed 38°C chloroform-methanol 2:1 (v/v) was added to the samples, mixed thoroughly and left to incubate for 5 min on ice. Samples were vortexed again and subsequently centrifuged at 14,000 × *g* for 10 min at 4°C. Three phases appeared and for lipid analysis the bottom organic phase was collected and transferred into a new reaction tube. Samples were air-dried in a Speedvac and pellets were reconstituted in 12 μL chloroform-methanol 2:1 (v/v). Samples were applied onto a thin layer chromatography silica gel 60G coated glass plate (Merck) and separated by using chloroform/methanol p.a./H_2_O (75%: 25%: 2.5%) as the solvent system. Lipids were visualized with 10% (w/v) phosphomolybdic acid in ethanol.

## Results

### Expression and Abundance of Flagella

**FIGURE 1:**
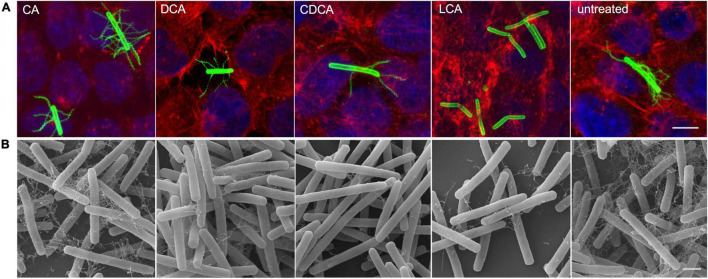
Flagella abundance. **(A)** Immunofluorescence (IF) of *C. difficile* challenged with the four different bile acids (CA, cholic acid; DCA, deoxycholic acid; CDCA, chenodeoxycholic acid; LCA, lithocholic acid) and untreated cells co-cultivated on Caco-2 cells. IF staining: green – anti-*C. difficile* antibody, red – Rhodamine-phalloidine and blue – DAPI, scale bar = 5 μm. **(B)** Scanning electron micrographs of exponential phase *C. difficile* cells challenged with BAs prior to co-cultivation with Caco-2 cells, scale bar = 1 μm.

**FIGURE 2:**
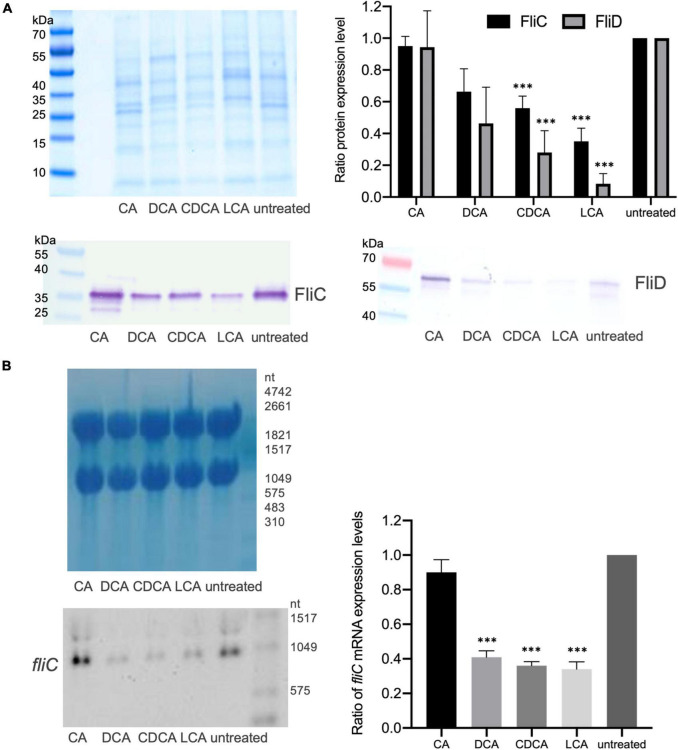
Flagella protein expression. **(A)** Western Blot analysis of FliC and FliD expression in untreated cells and in cells stressed with the four different BAs with a Coomassie-stained gel in the upper left corner that was run as a loading control in parallel. Right (top): Average quantification of signal intensities of FliC as well as FliD bands of three replicates normalized to untreated condition. **(B)** Transcriptional profiling of *fliC* expression by Northern Blotting. Left (top): Methylene blue staining of RNA gel serving as a loading control. Left (bottom): Exemplary *fliC*-specific Northern Blot. Right: Three biological replicates were analyzed and normalized to the control condition. Standard deviation is indicated and values significantly different by a Student’s *t*-test compared to untreated cells are marked (***p-value <0.001).

It was previously shown that distinct BAs have an impact on the presence of flagella on the cell surface of *C. difficile* influencing motility of the bacterial cell (Sievers et al., 2019). In the present work these data could be verified and extended. In Confocal Laser Scanning Microscopy (CLSM) and SEM analyses it was demonstrated that *C. difficile* cells challenged with CA feature just as many flagella as non-stressed bacteria. DCA and CDCA stress result in a reduced number of flagella while LCA challenge leads to an almost complete loss of flagella (Figures 1A,B). This is in perfect accordance with the data in our previous work, where flagella abundance was exactly quantified (Sievers et al., 2019). It should be noted that the different BA concentrations used in this study were adapted to provide a comparable extent of growth inhibition of *C. difficile*. The question remained, if the reduced number of flagella is caused by the sole loss of the filaments from the cell surface during sample preparation for microscopic analyses or if the cellular synthesis of flagellar components is influenced by BA. To give an answer, antibodies against FliC and FliD, both representing flagellar proteins, were used to determine the abundance of these two proteins on the cell surface of *C. difficile*. Extracts of *C. difficile* surface proteins were prepared, the proteins separated *via* SDS-PAGE and FliC and FliD quantified by Western Blot analysis. Western Blot signals reflect a lower abundance of flagella proteins in the presence of DCA and CDCA, and even more pronounced in the presence of LCA (Figure 2A), once more indicating a reduced flagella synthesis during BA stress with the exception of CA. In a next step, it should be clarified if differences in flagella abundance have their origin already on transcriptional level. For this reason, *fliC* mRNA abundance after challenge with different BAs was assayed in Northern Blot analysis (Figure 2B). The mRNA abundance resembles protein levels meaning that the lower expression of flagellar proteins, specifically FliC, during LCA, DCA, and CDCA challenge, is already implemented on transcriptional level.

### Adhesion to Epithelial Cells

**FIGURE 3:**
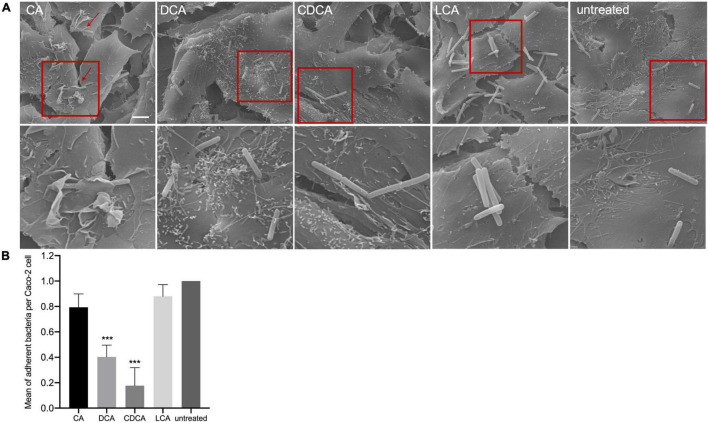
Co-cultivation of bile acid stressed *C. difficile* with Caco-2 cells. **(A)** Scanning electron micrographs of co-cultivation of Caco-2 cells with untreated *C. difficile* and *C. difficile* challenged with four different bile acids (CA, cholic acid; DCA, deoxycholic acid; CDCA, chenodeoxycholic acid; LCA, lithocholic acid), scale bar = 5 μm. Red squares indicate zoomed in area shown below. **(B)** Adhesion assay after co-cultivation of Caco-2 cells with bile acid stressed *C. difficile*. Colony forming units of three independent experiments with three technical replicates each were determined and the number of adherent bacteria per Caco-2 cell was visualized. Standard deviation is indicated and values significantly different by a Student’s *t*-test compared to untreated cells are marked (***p-value <0.001).

**FIGURE 4:**
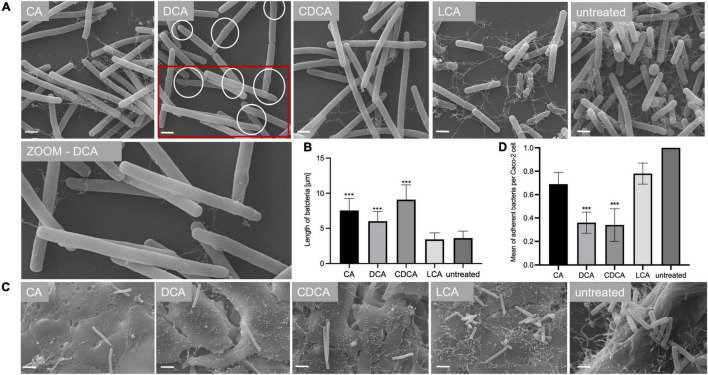
Morphology of bile acid stressed *C. difficile* in stationary phase. **(A)** Scanning electron micrographs of untreated *C. difficile* (untreated) and *C. difficile* challenged with four different bile acids (CA, cholic acid, DCA, deoxycholic acid; CDCA, chenodeoxycholic acid; LCA, lithocholic acid). Scale bar = 1 μm. Circles in DCA micrograph indicate “swellings.” **(B)** Average length of bacterial cells is given in μm. **(C)** Scanning electron micrographs of co-cultivated untreated *C. difficile* and *C. difficile* challenged with four different bile acids (CA, DCA, CDCA, and LCA) with Caco-2 cells, scale bar = 2 μm. **(D)** Adhesion assay after co-cultivation of Caco-2 cells with bile acid stressed *C. difficile* from stationary phase. Colony forming units of 3 independent experiments with three technical replicates each were determined and the number of adherent bacteria per Caco-2 cell was visualized. Standard deviation is indicated and values significantly different by a Student’s *t*-test compared to untreated cells are marked (***p-value <0.001).

In a previous study it was shown that BAs can cause a reduced motility of *C. difficile* with LCA-stressed cells being essentially immotile (Sievers et al., 2019). In this work, the ability of BA-challenged *C. difficile* cells to adhere to intestinal epithelial cells (Caco-2 cells) was tested, since adherence is not only a key feature for the infection process but also known to be mediated by flagella (Tasteyre et al., 2001). As expected, and in accordance with flagella abundance data, adhesion of CA-stressed *C. difficile* cells is comparable to unstressed cells (Figure 3). Also anticipated was the decrease in adherence of *C. difficile* stressed with DCA and CDCA, but unexpected was the persistent adherence of *C. difficile* which was stressed with LCA. These cells are almost devoid of flagella and must mediate their adherence to Caco-2 cells by some kind of flagella-independent way. This data was confirmed by repeating the adhesion assay with stationary *C. difficile* stressed with the four bile acids (Figures 4C,D). In stationary phase LCA-treated *C. difficile* have regained flagella growth (Figure 4A) but the number of bacteria that adhere to the Caco-2 cells remains very similar to cells grown to exponential phase only (Figures 4D, 3B).

### Toxin Expression

**FIGURE 5:**
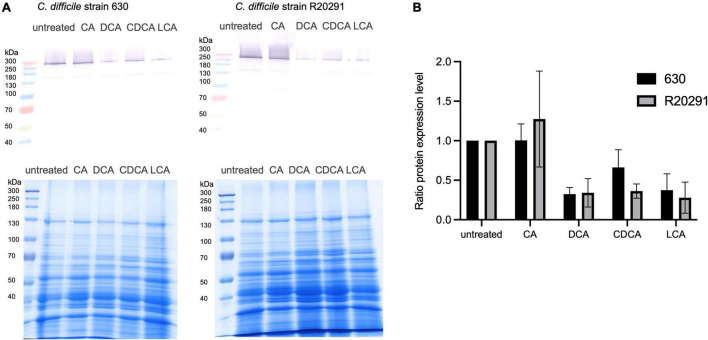
Western Blot analysis of secreted toxin A. **(A)** *C. difficile* strain 630 (left) and strain R20291 (right) were cultivated for 72 h in the presence of four different bile acids (CA, cholic acid; DCA, deoxycholic acid; CDCA, chenodeoxycholic acid; LCA, lithocholic acid) and without any stress conditions (untreated). Secreted proteins were extracted from cultivation supernatant and an amount of 150 μg was separated *via* SDS-PAGE (loading control in the bottom) to subsequently detect toxin A by Western Blot analysis (top). **(B)** Three biological replicates were analyzed and related to the untreated condition. Standard deviations are indicated.

It has been shown that flagella expression correlates with toxin expression in *C. difficile* (Aubry et al., 2012). Thus, we investigated if the presence of BAs has also an impact on the amount of toxins produced by *C. difficile*. The toxins are mostly synthesized in the late stationary phase with some strains producing more toxin than others (e.g., strain R20291). Growth media lacking specific nutrients, such as glucose and cysteine, even facilitate toxin expression (Bouillaut et al., 2015). In this study, *C. difficile* strains 630 and R20291 were grown for 72 h in tryptone medium containing different BAs. To measure toxin expression, the abundance of toxin A in the extracellular protein fraction was determined by Western Blot analysis (Figure 5 and Supplementary Figure 1). The amount of toxin A secreted by *C. difficile* into the medium correlates very well with flagella expression in exponentially growing cells stressed with different BAs. Both strains produce higher amounts of toxin in the absence of BAs. CA does not hamper toxin synthesis, but the other three BAs provoke a clear reduction of secreted toxin A.

### Cellular Location of Bile Acids

**FIGURE 6:**
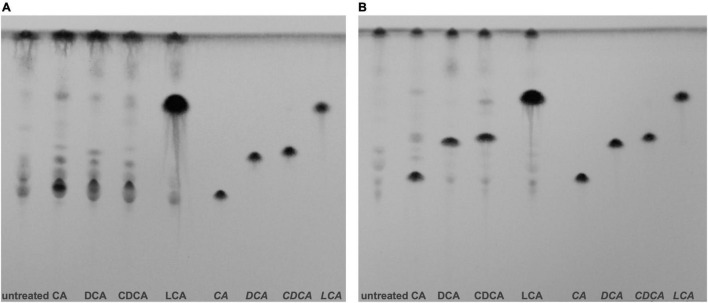
Thin layer chromatography (TLC) of *C. difficile* lipid extracts. **(A)** Lipids of exponentially growing cells and **(B)** stationary phase cells under control conditions (untreated) and challenged with different bile acids (CA, cholic acid; DCA, deoxycholic acid; CDCA, chenodeoxycholic acid; LCA, lithocholic acid) were extracted and separated *via* TLC. Hydrophobic species were stained with phosphomolybdic acid. References of the four bile acids were separated on the right side of each TLC plate. Two additional biological replicates for exponentially growing cells and stationary phase cells are provided Supplementary Figure 3.

Different BAs have different impact on *C. difficile*, not only on the expression of flagella, host cell adherence and toxin secretion as shown in this study, but also on germination, growth, biofilm formation and toxin action (Sorg and Sonenshein, 2008; Darkoh et al., 2013; Thanissery et al., 2017; Dubois et al., 2019). However, the molecular understanding for most of such differences is still missing. To elucidate the mode and place of action of BAs in the *C. difficile* cell, information on the cellular location of the single BAs is mandatory. There is no information yet on how efficiently external BAs from growth media or the gut lumen enter a *C. difficile* cell. In this study, we aimed for a first and semi-quantitative view in this regard. *C. difficile* cells stressed with growth-inhibiting concentrations of different BAs were obtained and their lipid fractions were extracted for a separation *via* TLC. Aside, pure BAs were applied on TLC plates and served as reference to pinpoint the single BAs on the plates. Although LCA was used in a much lower concentration compared to the other BAs, one can easily realize an extreme accumulation of LCA in *C. difficile* cells (Figure 6). This vast amount of LCA can already be detected in exponentially growing cells grown for 5 h in medium containing the BAs. In contrast, *C. difficile* cells challenged with DCA and CDCA show an enrichment in BAs first in stationary but barely in exponential growth phase. A clear accumulation of CA could be detected in stationary phase cells as well. The CA spot of the exponential growth phase sample overlays with signals of other lipids. Besides the enormous accumulation of LCA in *C. difficile*, differences can be noticed in lipid patterns comparing the different BA conditions. Especially in stationary phase, lipid composition of BA stressed cells appears to be different from untreated cells possibly caused by an adjustment of the cell membrane composition in response to BA stress.

**FIGURE 7:**
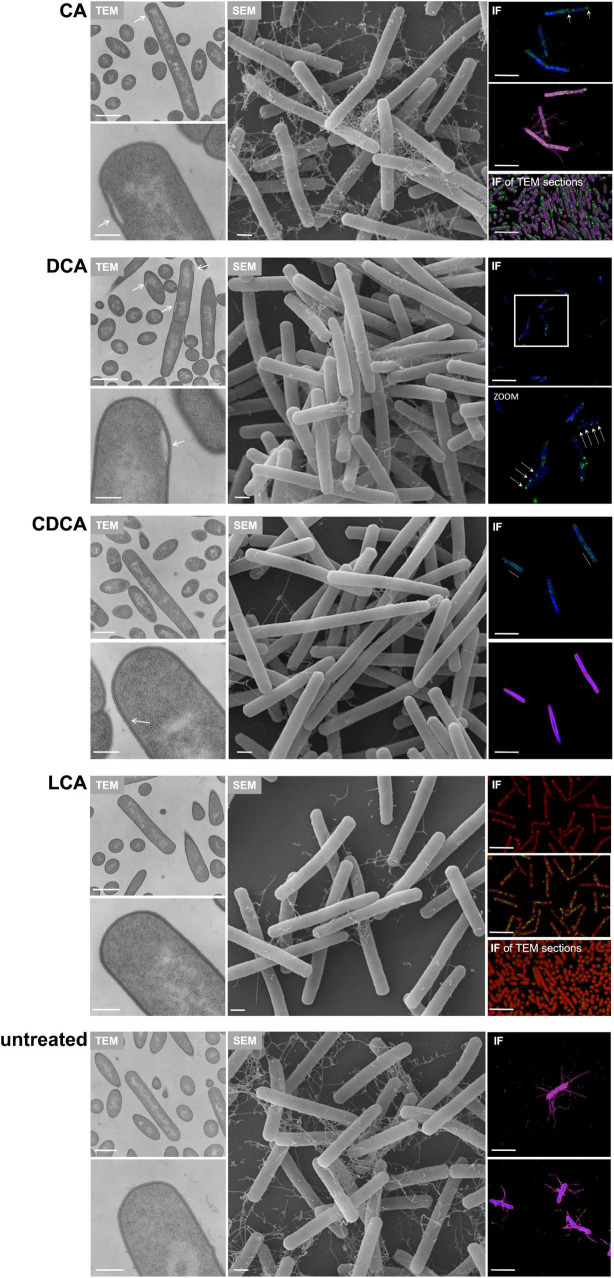
Putative localization of various BAs in the *C. difficile* cell. Left: Transmission electron micrographs of *C. difficile* challenged with the four different bile acids (CA, cholic acid, DCA, deoxycholic acid, CDCA, chenodeoxycholic acid, LCA, lithocholic acid) and of untreated cells, all in exponential growth phase. Morphological changes are highlighted as white arrows. Scale bars, top 1 μm and bottom 200 nm. Middle: Scanning electron micrographs of *C. difficile* challenged with four different bile acids (CA, DCA, CDCA, and LCA) and of untreated cells, scale bar = 1 μm. Right top and center: Immunofluorescence (IF) of *C. difficile* challenged with BAs and stained with mouse anti-CA antibody (green), mouse anti-CDCA antibody (green), rabbit anti-DCA antibody (green), rabbit anti-LCA antibody (green) as well as DAPI (blue) and rabbit anti-*C. difficile* antibody (purple). Due to the same antibody host (rabbit), LCA samples were not counterstained with anti-*C. difficile* but with Nile red (red) and DCA challenged cells were only co-stained with DAPI. Scale bars = 5 μm. Right bottom: IF of TEM sections of *C. difficile* challenged with BAs and stained with anti-CA (green) and counterstained with anti-*C. difficile* (purple) and anti-LCA (green) counterstained with Nile red (red). An IF staining of TEM sections of DCA, CDCA and untreated samples was not possible. Scale bars = 5 μm.

In addition to TLC experiments, microscopic methods were consulted to gain evidence on the cellular location of BAs. TEM revealed interesting differences on the impact of BAs in *C. difficile* (Figure 7). Especially, in DCA-challenged cells a formation of bleb-like structures (“swelling”) on the inner surface layer of the cell is most striking. Not as prevalent but also observable those swellings occur in CA-stressed cells. In untreated and LCA-stressed *C. difficile* none of such structures could be seen. Interestingly, in cells challenged with CDCA no swellings, but a distinct additional layer on the inner surface of the bacterial membrane was observed. In SEM of exponentially growing *C. difficile* cells stressed with different BAs these morphological changes were not visible, but differences in the expression of flagella became once more obvious (Figure 7). However, in SEM of stationary phase cells being challenged with BAs for a longer time, the swellings of DCA-stressed cells could be visualized (Figure 4). Furthermore, big differences in the length of bacteria were determined as was previously shown for exponentially growing cells (Sievers et al., 2019). Especially CDCA causes elongation with cells being twice as long as untreated stationary phase cells (Figure 4). However, most striking was the recurrence of flagella in LCA-stressed stationary phase cells, whereas *C. difficile* grown to stationary phase in the presence of DCA or CDCA still features a reduced number of flagella (Figure 4).

Besides electron microscopy we also employed CLSM to test for the fate of different BAs in *C. difficile* (Figure 7). This was possible since specific antibodies against each of the four tested BAs are commercially available. A proof of specificity and absence of cross-reactivity is provided in Supplementary Figure 2. Although resolution of the applied CLSM method is the limiting factor for an unequivocal determination of the exact cellular location of the single BAs, the results essentially resemble the TEM data. The immunofluorescence (IF) signal of the DCA antibody appears in separate spots apparently at the bacterial membrane. Such fluorescence spots could resemble the swellings seen in electron micrographs. Also in CA-challenged *C. difficile*, the IF signal originating from the BA antibody appears clustered but not distributed all over the cell. This becomes especially obvious when CA is detected *via* IF in an ultramicrotome section as it was prepared for TEM (Figure 7). In contrast, in cells challenged with CDCA the IF signal is not as focused but rather evolving from the entire cell envelope which corresponds very well with the phenomenon of an extra layer at the inner side of the cytoplasmic membrane as it was observed *via* TEM. Although, no abnormality was detected in electron micrographs of LCA-stressed cells, IF revealed an accumulation of LCA in several clusters over the cell comparable to what was observed for DCA.

## Discussion

Previously, comprehensive proteome signatures characterizing the stress response of the human intestinal pathogen *C. difficile* to the four most abundant BA lead structures have been published. They showed different and specific changes in *C. difficile’s* proteome depending on the nature of the BA (Sievers et al., 2018). Data of this study demonstrate that the expression of several virulence traits of *C. difficile* is diversely affected in the presence of various BAs as well. Our data revealed the close to complete loss of flagella in LCA challenged *C. difficile* cells and reduced number of flagella in cells cultivated in the presence of DCA and CDCA. The latter observation correlates very well with the observation made by Dubois et al. who determined an increased biofilm formation for DCA and CDCA stressed cells (Dubois et al., 2019), which could be reasoned by less motile cells with fewer flagella. Flagella play an important role for the pathogenicity of *C. difficile*. They do not only allow for motility of the bacterium but also mediate adhesion to biotic and abiotic surfaces (Stevenson et al., 2015). The here presented results on the reduced adherence of *C. difficile* challenged with DCA and CDCA to Caco-2 cells, a cell line of human colorectal adenocarcinoma cells, was thus expected. However, astonishing was the consistent adherence capability of LCA-challenged *C. difficile* cells, although earlier studies demonstrated the importance of flagella in *C. difficile* colonization (Tasteyre et al., 2001). Cells of *C. difficile* 630 tested in the present study, that lost flagella after LCA treatment, were obviously able to adhere to Caco-2 cells by a flagella-independent mechanism. This finding corresponds very well to recent observations in which strain 630Δ*erm* mutated in the structural flagella components *fliC* and *fliD* featured an even stronger adherence to Caco-2 cells than the wildtype (Dingle et al., 2011; Baban et al., 2013), but is contradictory to results obtained in strain R20291, in which the loss of flagella resulted in a decreased adherence to Caco-2 cells (Baban et al., 2013). The origin of such differences in adherence between strains and the mechanistic background of a flagella-independent cell adhesion still needs to be elucidated.

Flagella are not just important virulence determinants in terms of motility and cell host adherence of a pathogen. It is known that they are highly immunogenic with the ability to modulate the host immune response by triggering proinflammatory cytokines which is mediated by the Toll-like receptor 5 (Hayashi et al., 2001), also shown for *C. difficile* (Yoshino et al., 2013). Hence, *C. difficile* strains devoid of flagella can possibly hide away from clearance by the immune system and infect the host even more successfully than strains expressing flagella (Dingle et al., 2011). The here determined differential impact of various BAs on flagella expression could thus make a big difference on how *C. difficile* is recognized by the host immune system along the intestinal tract. Higher concentrations of DCA, CDCA and LCA, that are generally thought to counteract a *C. difficile* infection, reduce flagella on the bacterial surface and thus might prevent a fast clearance of the pathogen by the immune system.

In this work, we could show that the non-existence of flagella is not just caused by mechanical loss from the cell surface due to presence of specific BAs, but a result of changes in gene expression, specifically gene transcription. Hence, BAs either enter the cells and directly hamper transcription of flagellar genes or they have their locus of action somewhere else in the cell, but the signal is transduced to affect *fliC* gene transcription. Whatever mechanism holds true, it is interesting that the tested BAs act differently in this respect. The regulation of flagella expression in *C. difficile* is not fully understood. However, the genes encoding for numerous components to build the flagellar apparatus are located in three adjacent genetic regions and are divided into early- and late-stage flagellar genes, allowing for their hierarchical expression. Expression of the late-stage flagellar genes, such as the main structural flagellar component *fliC* and the flagellar cap *fliD*, is facilitated by sigma factor FliA (SigD), which itself is encoded in the region for early-stage flagellar genes (Aubry et al., 2012). These early-stage genes have recently been shown to be under control of a genetic switch upstream of their coding sequences deciding on their “ON” or “OFF” state and consequently, *via* SigD, also on late-stage flagellar gene expression (Anjuwon-Foster and Tamayo, 2017). Interestingly, the locus of early-stage flagellar genes is additionally regulated by a riboswitch located in the untranslated 5′ region of the corresponding mRNA. Binding of c-di-GMP to the riboswitch leads to premature termination of transcription (Soutourina et al., 2013). In our work, we detected a reduced amount of the late-stage flagellar proteins FliC and FliD and also of the *fliC* mRNA in the presence of the secondary BAs DCA and LCA and also of the primary BA CDCA. Several groups have already demonstrated a co-regulation of flagella and genes of the pathogenicity locus (PaLoc) including the two main toxins, toxin A and toxin B. Aubry et al. (2012) have shown an increased toxin production of *C. difficile* when the *fliC* or *fliD* gene is deleted accompanied by an increased virulence of such mutants in a hamster model. Our observation is the opposite with reduced levels of secreted toxin A comparable to the influence of the single BAs on *fliC* and *fliD* expression. A reduction of toxin synthesis was previously observed when genes of the early-stage flagellar locus are deleted (Aubry et al., 2012) leading us to the conclusion that the reduction of the late-stage flagellar components FliC and FliD and of toxin A, as determined in this work, is an indirect effect that results from repression of the early-stage flagellar operon. Specifically, the reduced amount of SigD, which is required for transcription initiation of both, the late-stage flagellar genes and the PaLoc (El Meouche et al., 2013) is assumed to be the cause of lower *fliC*, *fliD* and toxin A expression. We thus conclude that the BAs DCA, CDCA and especially LCA hamper expression of the early-stage flagellar gene region. Hitherto, BAs were known to affect spore germination and vegetative growth of *C. difficile*. Our observation endows them with another vital feature, i.e., the influence on the expression of important virulence traits such as flagella formation and toxin synthesis. Thus LCA, DCA and CDCA might counteract a *C. difficile* infection by suppressing transcription of early-stage flagellar genes and subsequently transcription of toxins, which once more underscores how the BA-composition in the host’s intestines can decide on the outcome of a CDI. A plethora of regulatory cellular processes to control the expression of the PaLoc in *C. difficile* has been described involving various sigma factors, other global transcriptional regulators and small metabolites (Martin-Verstraete et al., 2016). We propose the regulation of toxin synthesis by specific BAs to be indirect and mediated by SigD. However, it cannot be excluded that other regulatory mechanisms play a role that directly affect toxin expression after BA challenge or act indirectly *via* the expression of flagellar genes. Hence, the question on the exact molecular mechanisms of how BAs specifically modify transcription of flagellar and toxin genes remains to be answered and will be a challenging task for future studies.

An important issue that has been neglected in research so far is the question of the place and mode of action of BAs in bacteria. Of course, it is assumed that due to their amphiphilic and soap-like character, BAs primarily challenge cell envelope integrity. Stressing bacteria with high concentrations of BAs in *in vitro* studies will at a certain point simply lead to abrupt cell lysis. However, in addition to the membrane-disturbing effect, BAs were also reported to structurally alter other macromolecules as RNA, DNA and proteins (Begley et al., 2005). LCA was even reported to cause single strand breaks in mouse lymphoblasts (Kulkarni et al., 1980). In contrast to SDS or other chemical detergents represent BAs naturally occurring compounds. Especially with respect to intestinal bacteria they occur in concentrations that will not kill the bacterium, but will challenge it and will result in adaptation processes and stress responses, or will have certain signaling function to implement specific virulence mechanisms. The BA taurocholate as trigger for spore germination of *C. difficile* is a good example of such a signaling function with an essential meaning for the virulence of the pathogen (Sorg and Sonenshein, 2008). However, it is neither known to date, how much of externally applied BAs actually enter the bacterial cell to be effective there, nor what the exact mode of action on the bacterial surface, specifically in the cytoplasmic membrane, is. Since it is widely appreciated by now, that specific BAs can be beneficial to avoid a CDI while others are rather detrimental, one aim of this study was to get an idea on how efficiently different BAs enter the cell. The concentration of every BA used in this study was adjusted in such a way that the effect on *C. difficile* growth was comparable, as described before (Sievers et al., 2018). This should prevent any indirect and growth-dependent effects on flagella formation, adhesion and toxin synthesis that were also analyzed in this study. But it meant that the concentrations of CDCA, DCA and especially CA that were used to stress *C. difficile* were much higher than the one of LCA. It can be assumed that the effect amphiphilic substances can exert on bacteria highly depends on their capability of entering the cells. With respect to BAs, the fewer hydroxy-groups a BA has, the more hydrophobic it is and the more efficiently it embeds into or even crosses membranes (Schubert et al., 1983; Cabral et al., 1987; Kamp et al., 1993). This was shown on model membranes that consisted of only one or a few different lipids. Later it was found that the membrane-crossing ability of BAs does not only depend on the hydrophobicity of the BA but also on the lipid composition of the membrane (Hofmann et al., 2001). Proteins embedded in the membrane, as it is the case in real biological membranes, would add another factor of impact on membrane intercalation of BAs. To exactly answer the question of how well an amphiphilic substance interacts with or crosses the cellular membrane of a bacterium, the substance needs to be tested on the specific bacterium itself. There is only very little known on the membrane composition of *C. difficile*. A first phospholipid profiling of membranes of 11 different *C. difficile* strains was carried out by Drucker et al. (1996). Later, plasmalogens, which are ether lipids restricted to strictly anaerobic organisms and animals, and cardiolipin, a rigid lipid composed of a phosphatidylglycerol dimer, were identified in *C. difficile* membranes (Guan et al., 2014). However, information on *C. difficile*’s lipidome is still scarce and to our knowledge no lipidomics analysis tracking the changes in membrane lipid composition with changing conditions has been performed. For a start we aimed to determine the relative amount of the different BAs that were taken up or adsorbed by *C. difficile* using thin layer chromatography (TLC). Although this approach is only semi-quantitative, a clear difference could be observed between the single BAs. LCA, which was provided in only very small quantities to the medium compared to the other BAs, was taken up in huge amounts already in exponential growth phase. In fact, there is no further increase in LCA noticeable in stationary phase, i.e., LCA can obviously enter *C. difficile* very fast and efficiently. On the contrary, there is no clear signal of the other three BAs in exponential growth phase, although for CA this cannot be unambiguously stated, since there is an accumulation and overlay of several lipids on the TLC plate at the height of CA. In general, the lipid pattern of CA, DCA, and CDCA stressed cells is slightly different from control cells in exponential phase, indicating that *C. difficile* already adapted its membrane lipid composition. This difference comes even clearer in stationary phase cells. Now, a clear accumulation of CA, DCA and CDCA in *C. difficile* can be observed, although not as dominant as for LCA challenged cells. Furthermore, the signals of lipids apart from the BAs show distinct patterns for CA, DCA, and CDCA stressed cells. *C. difficile* cells challenged with LCA show the most similar pattern to the control sample despite the strong accumulation of LCA. Altogether, TLC results indicate that there is not just a proteomic stress response of *C. difficile* specific for every tested BA (Sievers et al., 2018), but that there are also BA specific rearrangements in the lipid composition of the pathogen.

As anticipated, the most hydrophobic BA LCA readily entered the *C. difficile* cell, but the other three BAs featuring more hydroxy groups were first detectable after a long incubation time. However, DCA and CDCA, which have the same empirical molecular formular, have a different impact in the cell as determined by proteomics profiling (Sievers et al., 2018) and by the TLC results presented in this study. It will be most interesting to characterize particular and BA-specific lipid changes in qualitative and quantitative detail making use of modern lipidomics techniques. The question remains, if there exist specific receptor molecules in the *C. difficile* cell and in bacteria in general. Most BA stress experiments on bacteria were carried out using a complex BA mixture isolated from ox or pig. Thus, very few examples of the impact of specific BAs are published so far. However, contrary reports on how bacteria respond to BAs point at the presence of specific receptors and signal transduction pathways. For instance, BAs have been shown to upregulate the flagellar promoter in *Campylobacter jejuni* (Allen and Griffiths, 2001) and motility in *Vibrio cholerae* (Krukonis and DiRita, 2003) but in *Salmonella enterica*, flagellar synthesis and motility was reduced in the presence of BAs (Prouty et al., 2004) comparable with what we observed in *C. difficile*. In 2013, Francis et al. (2013) determined the spore surface protease CspC to be the specific receptor of taurocholate to induce spore germination of *C. difficile*. CspC is the first and only receptor identified so far. However, in consideration of the numerous specific BA receptors that have already been described in human (Fiorucci and Distrutti, 2015), additional receptors aside from CspC are conceivable.

While TLC was used in this study to determine if and to what extent BAs accumulate in *C. difficile*, we tried to determine the exact location of BAs by different microscopic approaches. Especially for DCA a clear concentration of this BA into a few bleb-like clusters in the cell could be observed by electron microscopy as well as by CLSM. Although DCA and CDCA differ only in the position of one single hydroxy group on the steroid scaffold, such an accumulation could not be seen for the latter one. CDCA in *C. difficile* seems to uniformly disseminate at the entire inner layer of the cell envelope. It can be speculated that the clustered accumulation of BAs in the cell might be an adaptation strategy of *C. difficile* to the stress, since such an accumulation of BAs in form of micelles would lead to an inhomogeneous distribution in the cell and thus to a reduction of the local BA concentration in the cell allowing metabolic processes still to take place. However, there remains the question why some BAs accumulate in blebs in *C. difficile* while others do not. It is tempting to reason that based on their hydrophobic character BAs have different abilities to interact with the specific structure of the membrane. It is long known that the membrane of eukaryotic cells is not a homogeneous compartment, but rather structured in domains that consist of high amounts of specific lipids such as cholesterol and proteins for signal transduction pathways (lipid rafts) (Lingwood and Simons, 2010). Recently, lipid raft equivalents were also discovered in bacteria. They were named functional membrane microdomains (FMM) and comprise highly active membrane segments including processes of signal transduction and transport (López and Kolter, 2010). These FMM are characterized by a higher concentration of rigid lipids and a special protein called flotillin or flotillin-like protein (Langhorst et al., 2005). Whereas in eukaryotes cholesterol represents the rigid lipid of lipid rafts, bacteria are not able to produce cholesterol and enrich other lipids in their FMM, e.g., cardiolipin which was already identified from *C. difficile* membranes (Guan et al., 2014).

Bile acids featuring a steroid structure are reminiscent of rigid lipids. It is thus conceivable, that the interaction of specific BAs with FMMs is different than with other parts of the bacterial membrane and also dependent on the nature of the BA. In a human carcinoma cell line, a change in membrane domains was detected after BA stress, and by using radio-labeled BAs a differential interaction of these BAs with membrane domains was determined (Jean-Louis et al., 2006). Also in baby hamster kidney cells, BAs reorganize membrane structure and stabilize membrane domains resulting in a non-receptor-mediated signaling in these cells (Zhou et al., 2013). These results turn BAs into potential regulators of cell signaling and they call for an elucidation of the exact signal cascades that are initiated in bacteria of the digestive tract including intestinal pathogens as *C. difficile*. The knowledge could help to shed light on observations that were recently made. Kang et al. (2019) reported an increased inhibitory effect of tryptophan-derived antibiotics on *C. difficile* in the presence of DCA and LCA but not CA. Although the authors could not explain this additive effect of selected BAs and antibiotics yet, it is possible, that it is caused by an impact some BAs have on membrane structure formation but others do not. Especially with regard to pathogenic bacteria, the perturbation of FMM possibly leads to a loss of essential virulence traits as it was impressively pictured for methicillin-resistant *Staphylococcus aureus* that could be re-sensitized for penicillin after FMM disruption (García-Fernández et al., 2017).

In the meantime, it is widely appreciated that intestinal BA composition can decide on the outcome of an infection with *C. difficile*. To smartly employ the intestinal microbiota and its ability to convert BAs in CDI prevention and therapy, detailed knowledge on the molecular action of BAs on pathogenic intestinal bacteria such as *C. difficile* is mandatory.

## Data Availability Statement

The original contributions presented in the study are included in the article/Supplementary Material, further inquiries can be directed to the corresponding author.

## Author Contributions

NM and SS designed all experiments of this study, analysed and interpreted the data and wrote the manuscript. LL and NM performed the TLC experiments. NL and NM prepared flagella extracts and performed Western Blot analysis to check for FliC and FliD protein abundance. DT and NM analyzed fliC mRNA expression by Northern Blot analysis. NM prepared the extracts for visualization by CLSM, TEM, and SEM and carried out the CLSMimage analysis. RS performed the electron microscopy studies. SD and NM performed cell culture experiments. SD, L-SP, and DT performed the preparation of extracellular proteins and Toxin A detection by Western Blot analysis. All authors contributed to the article and approved the submitted version.

## Conflict of Interest

The authors declare that the research was conducted in the absence of any commercial or financial relationships that could be construed as a potential conflict of interest.

## Publisher’s Note

All claims expressed in this article are solely those of the authors and do not necessarily represent those of their affiliated organizations, or those of the publisher, the editors and the reviewers. Any product that may be evaluated in this article, or claim that may be made by its manufacturer, is not guaranteed or endorsed by the publisher.

## Supplementary Material

Supplementary Material 1 Western Blot analyses of toxin A from supernatants of *C. difficile* (strains 630 and R20291) stressed with different bile acids and of unstressed cells.

Supplementary Material 2 Verification of specificity and exclusion of cross reactivity of four bile acid-specific antibodies.

Supplementary Material 3 Thin layer chromatography (TLC) of lipid extracts of *C. difficile* stressed with bile acids and of unstressed cells.
