# Supplementary figures and images for: Destination and Specific Impact of Different Bile Acids in the Intestinal Pathogen Clostridioides difficile
Source: Front Microbiol. 2022 Mar 24;13:814692. doi: 10.3389/fmicb.2022.814692 (PMC8989276; doi:10.3389/fmicb.2022.814692)

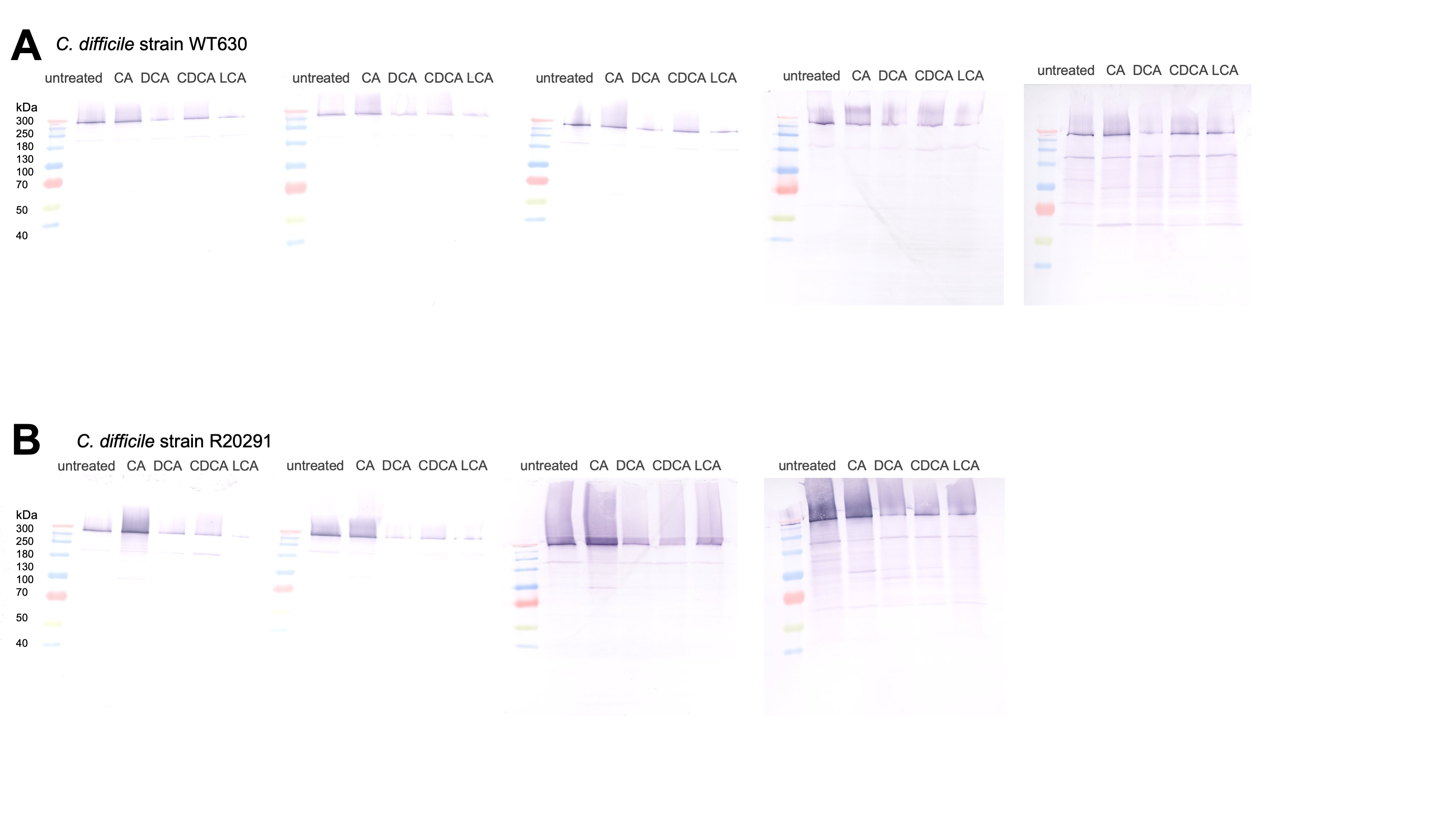

Supplement: Supplementary Material 1 — Western Blot analyses of toxin A from supernatants of C. difficile (strains 630 and R20291) stressed with different bile acids and of unstressed cells. [file Image_1.tiff]

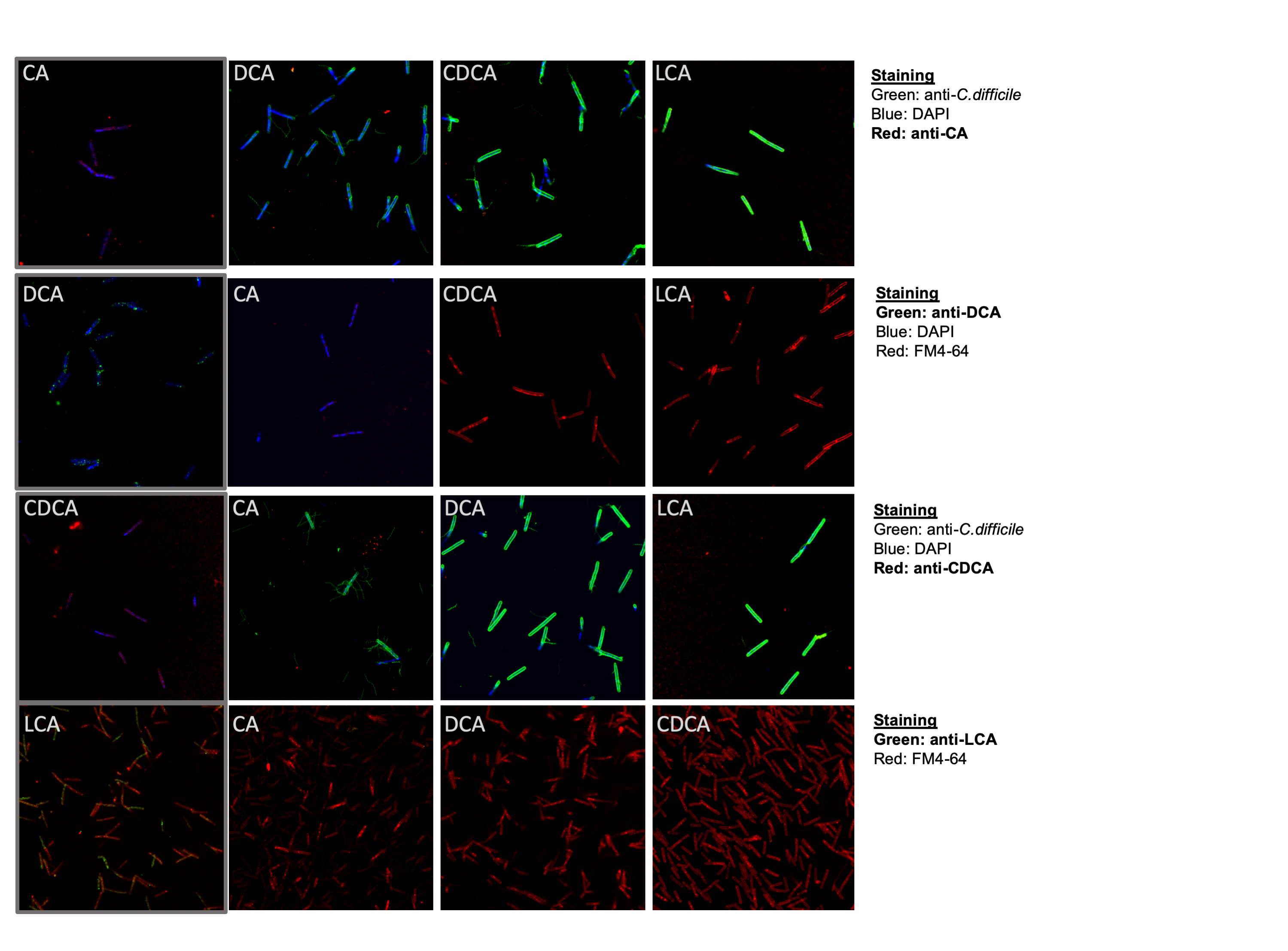

Supplement: Supplementary Material 2 — Verification of specificity and exclusion of cross reactivity of four bile acid-specific antibodies. [file Image_2.TIFF]

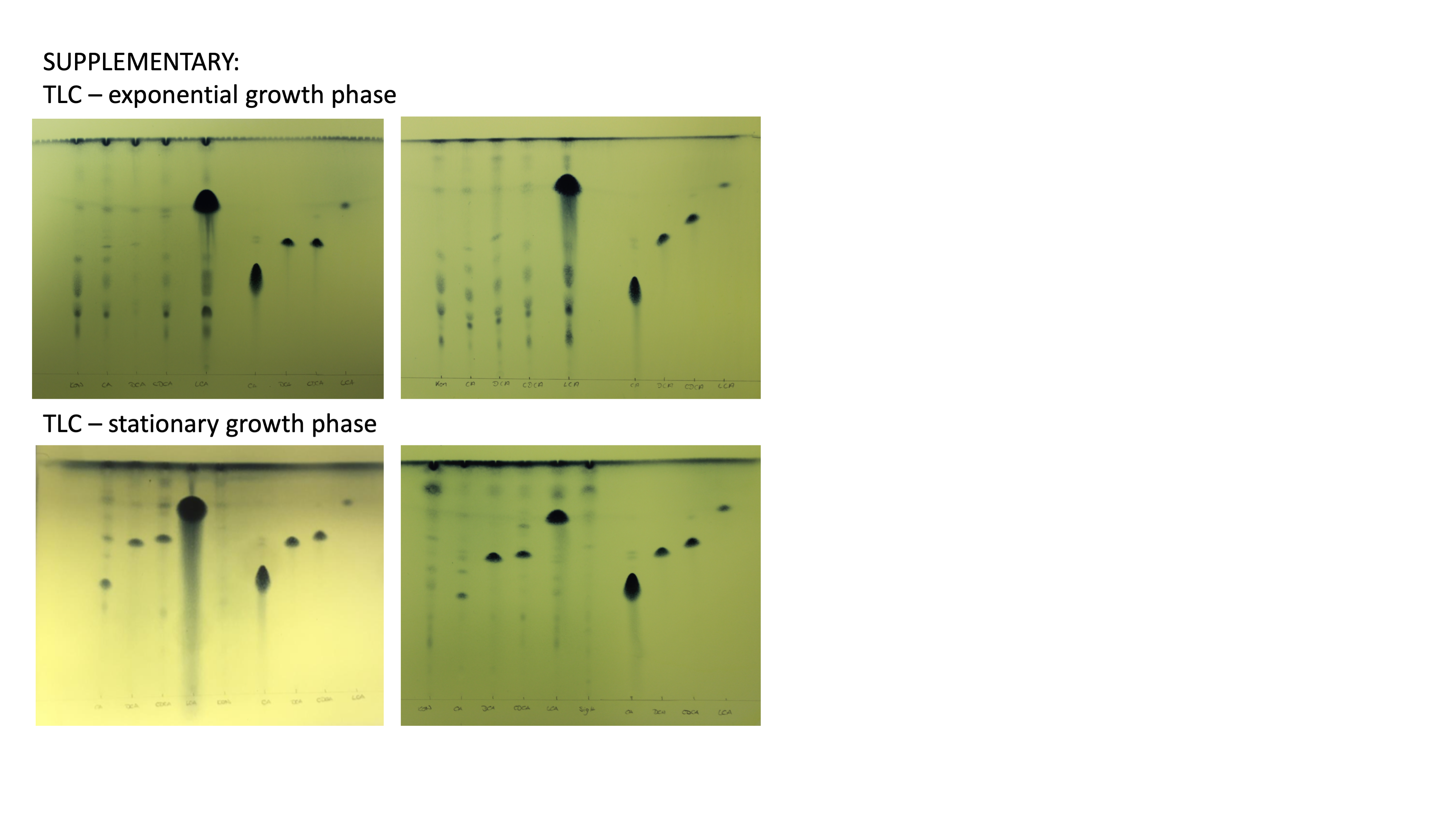

Supplement: Supplementary Material 3 — Thin layer chromatography (TLC) of lipid extracts of C. difficile stressed with bile acids and of unstressed cells. [file Image_3.TIFF]
